# Identification of resistance to *Fusarium* head blight and molecular cytogenetics of interspecific derivatives between wheat and *Psathyrostachys huashanica*

**DOI:** 10.1270/jsbbs.21089

**Published:** 2022-06-11

**Authors:** Chenchen Hou, Jing Han, Liangliang Zhang, Qiang Geng, Li Zhao, Shuhui Liu, Qunhui Yang, Xinhong Chen, Jun Wu

**Affiliations:** 1 Shaanxi Key Laboratory of Plant Genetic Engineering Breeding, College of Agronomy, Northwest A&F University, Yangling, Shaanxi, 712100, China

**Keywords:** *Fusarium* head blight, *Psathyrostachys huashanica*, ditelosomic addition line, substitution line, molecular cytogenetics, marker analysis, 660K genotyping array analysis

## Abstract

*Psathyrostachys huashanica* is a relative of wheat (*Triticum aestivum* L.) with many disease resistance genes that can be used to improve wheat disease resistance. In order to enrich the germplasm resources available in wheat genetics and breeding, we assessed *Fusarium* head blight (FHB) resistance in 45 interspecific derivatives between wheat and *Psathyrostachys huashanica* during two years from 2017–2018. Two interspecific derivatives comprising, H-34-8-2-6-1 and H-24-3-1-5-19-1 were identified as FHB resistant lines. These two lines were examined based on their morphology and cytogenetics, as well as by genomic *in situ* hybridization (GISH), fluorescence *in situ* hybridization (FISH), molecular markers, and 660K genotyping array to determine their genetic construction. The results confirmed H-34-8-2-6-1 as a wheat–*P. huashanica* 1Ns long arm ditelosomic addition line and H-24-3-1-5-19-1 as a wheat–*P. huashanica* 2Ns substitution line. Assessments of the agronomic traits showed that H-34-8-2-6 had significantly higher kernel number per spike and self-fertility rate than parent 7182. In addition, compared with 7182, H-24-3-1-5-19-1 had a much lower plant height while the other agronomic traits were relatively similar. The two new lines are valuable germplasm materials for breeding FHB resistance in wheat.

## Introduction

*Fusarium* head blight (FHB), also known as scab, pink mold, and whiteheads, is caused by *Fusarium graminearum* that affects wheat heads, and occurs widely in humid and semi-humid regions throughout the World (Abaya *et al.* 2021, Goswami and Kistler 2004, Osborne and Stein 2007). Due to global warming and wheat planting structure changing, FHB has became a world-wide disease in wheat and it has caused severe losses in the United States, Canada, Europe, and South America (McMullen *et al.* 1997, Tan *et al.* 2004). In China, FHB mainly affects the winter wheat producing regions located in the middle-lower reaches of the Yangtze River and South China, and the spring wheat producing regions of Northeast China (Lu *et al.* 2008). In recent years, the severity of FHB has increased in the wheat producing regions located in the Huang Huai River, and Northwest China. FHB causes significant reductions in the wheat yield as well as decreasing the starch and protein contents of wheat kernels. Most importantly, the blighted wheat kernels contain deoxynivalenol secreted by *Fusarium*, which detrimentally affects the health of humans and animals (Desjardins and Hohn 1997, Duveiller *et al.* 2008, Gunupuru *et al.* 2017, Krska 2009). Chemical and biological control methods are effective against FHB, but threaten both environmental safety and food safety. Therefore, breeding FHB resistance in wheat is a fundamental approach for controlling FHB and also one of the important goals in wheat breeding.

Many studies have investigated FHB, but there is a lack of FHB-resistant varieties of wheat scab in China and abroad (Chang *et al.* 2015). The only major FHB resistance sources at present are Sumai 3 and Wangshuibai, and their closely related derived lines. The main previously identified resistance genes are *Fhb1* (Cuthbert *et al.* 2006) and *Fhb2* (Cuthbert *et al.* 2007) in Sumai 3, and *Fhb4* (Xue *et al.* 2010) and *Fhb5* (Xue *et al.* 2011) in Wangshuibai. However, these resistant sources generally exhibit poor performance in terms of their yield, baking quality, and adaptability (Bai and Shaner 2004). Compared to Sumai 3 and Wangshuibai, using FHB resistance from wheat-alien lines is a challenge. The identification of three major resistant genes comprising, *Fhb3* in *Leymus racemosus* (Qi *et al.* 2008), *Fhb6* in *Elymus* (Cainong *et al.* 2015), and *Fhb7* in *Elytrigia elongata* (Guo *et al.* 2015), motivated the search for FHB resistance in close relatives of wheat. Thus, the identification and exploitation of FHB resistance genes in close relatives of wheat may facilitate breeding new FHB-resistant varieties of wheat.

In recent years, many genera that are closely related to wheat have been shown to possess FHB resistance, and the introduction of FHB resistance genes into wheat has been investigated by distant hybridization and studies of the resultant interspecific derivatives. For example, the FHB-resistant ditelosomic substitution line 7Lr#1S(7A) were bred by introducing the Chromosome 7Lr#1S of *Leymus racemosus* into common wheat (Wang and Chen 2008). A wheat-*Thinopyrum elongatum* 7E chromosome FHB-resistant disomic addition line was constructed by introducing the diploid *Thinopyrum elongatum* 7E chromosome into the wheat background which showed high resistance to FHB (Liu *et al.* 2017a). The new wheat varieties comprising Xinong 509, Xinong 511, and Xinong 529 derived from the common wheat–decaploid *Elytrigia elongata* line have been shown to exhibit good FHB resistances in the field (Liu *et al.* 2017b). In addition, a wheat–*Leymus racemosus* translocation line T6DL·7LrS provided a new germplasm for genetic improvement of wheat scab (Zhang and Wang 2018).

*Psathyrostachys huashanica* is closely related to wheat and it possesses many genes for beneficial traits that can improve wheat germplasm, such as resistance to drought, chilling and freezing, high salinity and high alkalinity, as well as resistance to diseases including wheat stripe rust, leaf rust, powdery mildew, and FHB (Chen *et al.* 1991, Zhao *et al.* 2010). Moreover, it can be crossed with wheat to transfer resistance genes, thereby allowing breeders to successfully introduce beneficial genes from *P. huashanica* into common wheat to improve its disease resistance (Chen *et al.* 1991, Kang *et al.* 2011). For example, the wheat–*P. huashanica* 1Ns disomic addition line H5-5-4-2 had immunity to powdery mildew at the seedling and adult stages (Han *et al.* 2020). In addition, the wheat–*P. huashanica* 2Ns, 3Ns, 4Ns, and 5Ns addition lines are highly resistant to wheat stripe rust, while the wheat–*P. huashanica* 1Ns and 7Ns addition lines are highly resistant to brown leaf rust, and the 2D(2Ns) substitution line 16-6 is highly resistant to wheat stripe rust (Du *et al.* 2013a, 2013b, 2014a, 2014b, 2014c, 2014d, 2015). However, there have been no previous reports of FHB resistance in the interspecific derivatives between wheat and *P. huashanica*.

## Materials and Methods

### Materials

The materials comprised *P. huashanica* (2n = 2x = 14, NsNs), wheat 7182 (2n = 6x = 42, AABBDD), and 45 interspecific derivatives between wheat and *P. huashanica* which are generated by taking 7182 as a female parent and *P. huashanica* as a male parent for hybridization, backcrossing with 7182 for one generation, and finally selfing for nine generations. The parental wheat 7182 were used as controls for assessing FHB resistance. These materials were developed and maintained by the Provincial Key Breeding Lab of Shaanxi. The FHB inoculum was provided by Northwest Agriculture and Forestry University (NWSUAF) College of Plant Protection.

### FHB resistance assessment of phenotyping

The 45 interspecific derivatives between wheat and *P. huashanica* were planted at Guancun Farm of NWSUAF in 2017 and 2018. The lines were manually inoculated with *F. graminearum* in the wheat blooming stage by glume cutting and drip inoculation. The inoculated FHB strain was rotationally cultured in a triangular flask containing 4% Mung bean (*Vigna radiata* L.) solution at 25°C for 2 days. The inoculum was diluted with sterile water until about 20 conidia were counted by microscopy in a hemocytometer at 10× magnification. Next, two symmetrical spikelets on one randomly selected spike were inoculated with 10 μL of inoculation solution after cutting the glumes, and the spike was then bagged to maintain the humidity for two days. Five plants were inoculated in this manner for each line. At 21 days after inoculation, the FHB symptoms were classified according to the ‘Rulers for Resistance Evaluation of Wheat to Diseases and Insect Pests Part4: Rule for Resistance Evaluation of Wheat to Wheat Scab’ (NY/T1443.4-2007) (Institute of Plant Protection 2007) (Supplemental Table 1).

The resistance and susceptibility to FHB were defined in terms of the disease index according to the ‘FHB Reviewing Standards’ as follows: resistant with a disease index below 20, moderately resistant with a disease index ranging from 20–40, moderately susceptible with a disease index ranging from 40–80, and highly susceptible with a disease index ranging from 80–100.

Disease index = Σ (spike number with all resistance and susceptibility grades × number with corresponding resistance and susceptibility grades)/(total number of spikes surveyed × maximum resistance and susceptibility grade) × 100

### Cytological observation

Seeds of the disease-resistant materials were spread on separate filter papers at the bottom of Petri dishes and soaked with water at room temperature for 24 h. The seeds were then arranged uniformly after removing the water and maintained in the Petri dishes until they were at the germination stage. The seeds were moved into a refrigerator and kept at 4°C for 24 h, before placing in dark until the roots grew to a length of 1–2 cm. The root tips were removed with tweezers and placed in ice water for 24 h; before fixing in Carnoy’s Fluid (glacial acetic acid:anhydrous ethanol = 1:3 v/v) for one week (Li *et al.* 2019). Then the root tips were placed in 2% cellulase plus 1% pectinase, hydrolyzed at 37°C for 1 h, ground, and centrifuged at 4000 rpm for 3 min. Finally, glacial acetic acid was added, before shaking and on a glass slide. Microsections were observed with an Olympus BX60 microscope to examine and count the numbers of chromosome structures, and images were acquired (Li *et al.* 2020c).

### Genomic *in situ* hybridization (GISH)

Cetyltrimethylammonium bromide (CTAB) was used to extract genomic DNA from the materials, the genomic DNA from *P. huashanica* was labeled with DIG-Nick Translation Mix (Roche, Germany) according to the manufacturer’s instructions. Clear microsections with multiple cell division phases were selected. To each of the microsections, 40 μL of hybridizing solution was added containing 4 μL probe DNA (at a concentration >1000 ng/μL), 1 μL DNA sodium salt from salmon testes (5 μg/μL), 4 μL 20× SSC (prepared from chloride sodium and trisodium citrate by mixing), 8 μL 50% (w/v) dextran sulfate, 20 μL formamide, and 1 μL 10% (w/v) sodium dodecyl sulfate, which were mixed, and ultrapure H_2_O was added to the solution until the volume reached 40 μL. The hybridization signals in the microsections were visualized with anti-digoxigenin fluorescein isothiocyanate. The microsections were stained with PI and then mounted in anti-fade agent H-1300, before examining with an Olympus BX60 microscope and capturing images using a Photometrics SenSys CCD (Wu *et al.* 2006).

### Fluorescence *in situ* hybridization (FISH)

Oligo-pSc119.2 (6-FAM-5ʹ) and Oligo-pTa535-1 (Tamra-5ʹ) as mixed probes were used to determine the chromosome constitution in H-24-3-1-5-19-1 (Li *et al.* 2020b). First, the glass slides were irradiated with 1250J light for 6min in UV Crosslinker. Second, 9 μL of mixed probe (pTa535-1:pSc119.2-2:H_2_O = 3:2:4) was added to the slide, and hybridized for 4 hours or overnight in a dark and humid environment at 37°C. Third, the microsections were stained with DAPI and10 μL of staining solution (H1200: ddH2O = 15:5) was added after drying. Last, observed and took pictures under an Olympus BX60 microscope.

### Expressed sequence tag-sequence tagged site (EST-STS) molecular marker analysis

Exogenous chromosomes were detected using 83 pairs of EST-STS primers (Han *et al.* 2020), which were evenly distributed on each chromosome in the seven wheat homologous groups. Whole genomic DNA was extracted with CTAB from 7182, H-34-8-2-6-1, H-24-3-1-5-19-1, and *P. huashanica*. After confirming the quality of the DNA by 1% agarose gel electrophoresis, the concentration was adjusted to 50 ng/mL for use as a PCR template. The PCR mixture with a volume of 20 μL contained 2 μL 10× PCR buffer, 2 μL (2.5 μmol/mL) primers, 2 μL (50 μg/μL) DNA template, 1.6 μL dNTPs (2.5 μmol/mL), 1.6 μL MgCl_2_ (2.5 mmol/mL), 0.1 μL (5 μmol/μL) *Taq* polymerase, and 10.7 μL deionized distilled water. The PCR products were subjected to electrophoresis on 8% non-denaturing polyacrylamide gel (voltage = 160 V, current = 120 mA) for 2.5 h, before observing in an ultraviolet light box and capturing an image with an SLR camera.

### Simple sequence repeat (SSR) molecular markers

222 pairs of SSR primers uniformly distributed on wheat chromosomes were used to determine the missing chromosomes in H-24-3-1-5-19-1 (Gupta *et al.* 2002, Pestsova *et al.* 2000, Röder *et al.* 1998, Zhang *et al.* 2008). The reaction system and PCR amplification procedure employed for analysis with the SSR markers were the same as those for the EST-STS markers, but the annealing temperatures differed for some primers. The remaining steps are the same as EST-STS.

### 660K genotyping array analyses

Whole genomic DNA was extracted using CTAB from H-24-3-1-5-19-1, 7182 and *P. huashanica*, and sent to Beijing CapitalBio Technology Co., Ltd. for analysis with Axiom 660K Genotyping Arrays. The heterozygous single nucleotide polymorphism (SNP) ratio and the specific SNP ratio in H-24-3-1-5-19-1 were calculated in Office Excel 2010 to determine the homologous group belonging to foreign chromosomes in H-24-3-1-5-19-1 (Bai *et al.* 2020).

### Agronomic traits of FHB-resistant materials

The agronomic traits of H-34-8-2-6-1, H-24-3-1-5-19-1 and 7182 were investigated after maturation. Ten plants from each of the interspecific derivatives and their parent common wheat 7182 were randomly selected to determine the plant height, spike length, tiller number per plant, spikelet and kernel numbers per spike, and kernel setting percentage per spike. The average data based on ten samples and two consecutive two-year repetitions were collected to ensure accurate results. Significant differences in these traits were determined with SPSS Statistics 20.

## Results

### Resistance to FHB

**Fig. 1.**
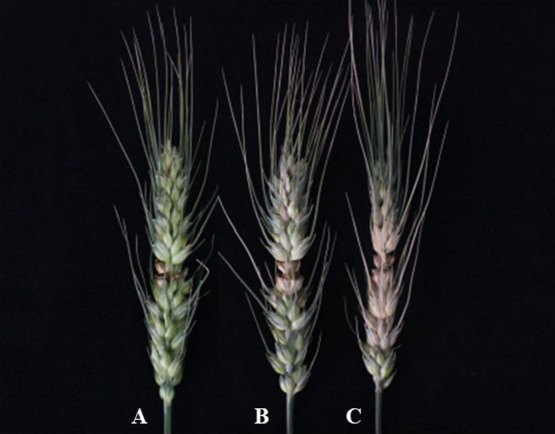
*Fusarium* head blight incidence in spikes. (A) H-34-8-2-6-1, (B) H-24-3-1-5-19-1, and (C) wheat 7182.

The disease grades and indexes for the 45 interspecific derivatives between wheat 7182 and *P. huashanica* were obtained in 2017–2018 (Table 1). Two of the 45 derived materials, H-34-8-2-6-1 and H-24-3-1-5-19-1, were moderately resistant to FHB (Fig. 1). The others were moderately susceptible to FHB, and the disease indexes for 7182 were 68 and 76 in 2017 and 2018, respectively, and thus it was moderately susceptible. The results suggested that moderate susceptibility was due to 7182, whereas the moderately resistant lines probably carried chromatin derived from *P. huashanica*.

### Cytological examination and GISH analysis of interspecific derivatives

**Fig. 2.**
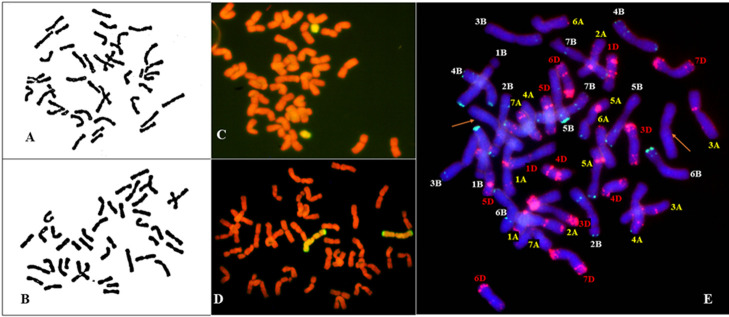
Cytogenetic identification of wheat–*P. huashanica* derivative lines H-34-8-2-6-1 and H-24-3-1-5-19-1. (A) Chromosomes in the root tip somatic cells of H-34-8-2-6-1 during mitotic metaphase (2n = 44). (B) Chromosomes in the root tip somatic cells of H-24-3-1-5-19-1 during metaphase mitosis, (2n = 42). (C) GISH analysis of H-34-8-2-6-1 using the total DNA from *P. huashanica* as the probe, and the wheat chromosomes were counterstained with PI (red). (D) GISH analysis of H-24-3-1-5-19-1 using the total DNA from *P. huashanica* as the probe, and the wheat chromosomes were counterstained with PI (red). (E) FISH analysis of the same metaphase after GISH analysis of H-24-3-1-5-19-1 by using pTa535 (red) and pSc119.2 (green) simultaneously as probes, and the wheat chromosomes were counterstained with DAPI (blue). Arrows indicate the pair of *P. huashanica* chromosomes.

The root tip chromosomes of H-34-8-2-6-1 and H-24-3-1-5-19-1 were observed during mitosis metaphase to determine the chromosome number. The results showed that the chromosome number of H-34-8-2-6-1 was 2n = 44 (Fig. 2A), and that of H-24-3-1-5-19-1 was 2n = 42 (Fig. 2B).

The root tip cells of the two lines in metaphase of mitosis were tested by GISH with Ns genomic DNA from *P. huashanica* as the probe and genomic DNA from 7182 as the blocker. The results showed that the root tip cells of H-34-8-2-6-1 contained two telocentric chromosomes with yellow-green hybridization signals, i.e., two additional chromosome arms from *P. huashanica* (Fig. 2C), thereby confirming that H-34-8-2-6-1 was a wheat–*P. huashanica* alien ditelosomic addition line. H-24-3-1-5-19-1 had two yellow-green chromosomes with 2n = 42 (Fig. 2D), and thus H-24-3-1-5-19-1 was a wheat–*P. huashanica* substitution line.

### Analysis with EST-STS molecular markers

**Fig. 3.**
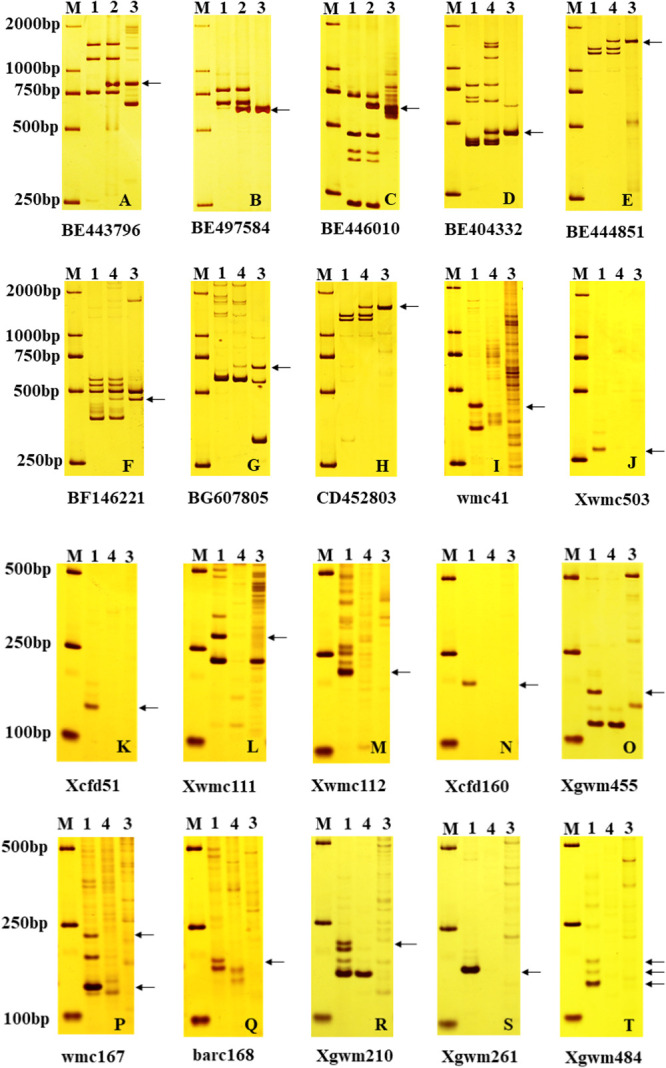
Amplification with specific molecular markers distributed in wheat homologous groups and wheat chromosomes. M: DNA ladder (DL2000); 1: 7182; 2:H-34-8-2-6-1; 3: *P. huashanica*; 4: H-24-3-1-5-19-1. Arrows of A to H indicate the specific amplification product from *P. huashanica*. Arrows of I to T indicate the specific amplification product from 7182.

83 pairs of EST-STS primers were used to identify the homologous groups of exogenous chromosomes in H-34-8-2-6-1 and H-24-3-1-5-19-1. Three EST-STS primers, BE443796, BE497584, and BE446010 (Supplemental Table 2) that mapped to homoeologous group I amplified clear *P. huashanica*-specific bands in line H-34-8-2-6-1 but none in 7182 (Fig. 3A–3C). However, the other primers did not amplify the specific bands of *P. huashanica*. Therefore, H-34-8-2-6-1 was considered to be a wheat–*P. huashanica* 1Ns long arm ditelosomic disomic addition line. Five pairs of EST-STS primers distributed in homoeologous group 2 of wheat amplified *P. huashanica*-specific bands in H-24-3-1-5-19-1 (Fig. 3D–3H), i.e., BE404332, BE444851, BF146221, BG607805, and CD452803 (Supplemental Table 2), but no specific bands from *P. huashanica* were amplified by other primers. Thus, the chromosomes from *P. huashanica* introduced into H-24-3-1-5-19-1 belonged to homoeologous group 2, confirming that H-24-3-1-5-19-1 was a wheat–*P. huashanica* 2Ns substitution line.

### SSR molecular marker analysis

222 pairs of SSR primers were used to determine the missing wheat chromosomes in H-24-3-1-5-19-1. Twelve pair of SSR primers distributed on 2D chromosome did not amplify products in H-24-3-1-5-19-1 and *P. huashanica*, but they amplified obvious specific alleles in 7182 (Supplemental Table 3, Fig. 3I–3T). Other primers amplified the same main amplicon in H-24-3-1-5-19-1 and 7182. These results indicate that a pair of 2D chromosomes from 7182 was missing in H-24-3-1-5-19-1, which preliminarily indicated that H-24-3-1-5-19-1 was a wheat–*P. huashanica* 2Ns(2D) substitution line.

### FISH analysis

Oligo-pSc119.2 (6-FAM-5ʹ) and Oligo-pTa535-1 (Tamra-5ʹ) were used as mixed probes to confirm the missing wheat chromosome in H-24-3-1-5-19-1. All chromosomes were present except for the 2D chromosome in H-24-3-1-5-19-1, thereby confirming that H-24-3-1-5-19-1 lacked a pair of 2D chromosomes (Fig. 2E), which further affirmed H-24-3-1-5-19-1 was a 2Ns(2D) substitution line.

### 660K genotyping array analyses

**Fig. 4.**
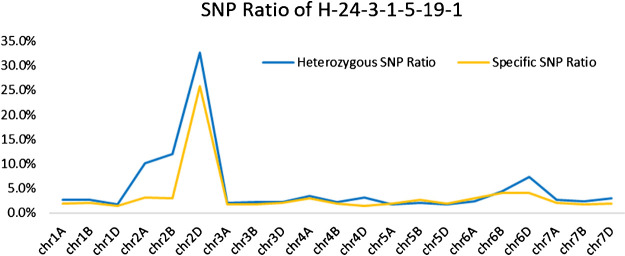
Wheat 660K SNP Array data analysis for H-24-3-1-5-19-1. The blue line represents the heterozygosity ratio for each chromosome in H-24-3-1-5-19-1 and the orange line represents the specific SNP ratio for each chromosome in H-24-3-1-5-19-1 relative only to *P. huashanica*.

Wheat Axiom 660K genotyping arrays were used for genotyping to further explore the chromosomal composition of H-24-3-1-5-19-1. The ratio of heterozygous genotypes on each wheat chromosome in H-24-3-1-5-19-1 was calculated. The results show that the ratio of heterozygous genotypes on the 2D chromosome (32.69%) was significantly higher than that on other wheat chromosomes (Fig. 4). In addition, we calculated the specific SNP ratio for each chromosome in H-24-3-1-5-19-1 compared with *P. huashanica* and found that the specific SNP ratio (25.82%) on the 2D chromosome in H-24-3-1-5-19-1 was significantly higher than that for the other chromosomes (Fig. 4). Therefore, we confirmed that the missing wheat chromosome was 2D and the *P. huashanica* chromosome in H-24-3-1-5-19-1 was 2Ns, and H-24-3-1-5-19-1 was a 2Ns(2D) substitution line, thereby validating the results obtained using EST-STS and SSR markers analyses, as well as by FISH analysis.

### Agronomic traits of resistant materials

**Fig. 5.**
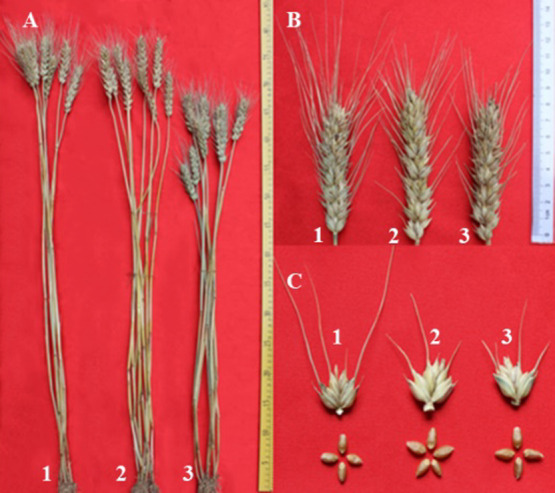
Agronomic traits of resistant materials. Plants (A), spikes (B), spikelets, and seeds (C) in H-34-8-2-6-1 (2), H-24-3-1-5-19-1 (3), and wheat parent 7182 (1).

The morphological characters determined for H-34-8-2-6-1, H-24-3-1-5-19-1, and their parent wheat 7182 (Fig. 5, Table 2) showed that H-34-8-2-6 had significantly higher kernel number per spike and self-fertility rate than 7182, and H-24-3-1-5-19-1 was much shorter than 7182 (p = 0.05 and 0.01). The other traits determined for H-34-8-2-6-1 and H-24-3-1-5-19-1 were similar to those in 7182.

## Discussion

FHB is a major disease that severely affects Triticeae crops throughout the world. The best approach for preventing the damage caused by FHB is breeding resistant varieties, which demands the selection and production of resistant sources (Shi *et al.* 2020). FHB resistances may be assessed differently among years and regions because of environmental influences, thereby hindering the screening of resistant offspring. Since the 1970s, Chinese researchers have successfully identified only two FHB-resistant materials comprising Sumai 3 and Wangshuibai among more than 30,000 test materials. However, long-term planting of with single resistance genes varieties will make the resistance ineffective, so new varieties are urgently needed to improve the FHB resistance of wheat (Gervais *et al.* 2003, Ruckenbauer *et al.* 2001). The numerous species related to wheat possess many excellent genes that are lacking in wheat and they can be used as valuable resources to improve the genetic basis for wheat disease resistance (He *et al.* 2007, Tester and Langridge 2010).

*P. huashanica* hybridizes well with common wheat. Many studies have shown that *P. huashanica* is an important material for distant hybridization with wheat because many resistance genes are present on its Ns chromosomes. Numerous disease resistance genes have been found in *P. huashanica* in recent years (Li *et al.* 2012, 2020a). However, the resistance to FHB by the interspecific derivatives between wheat and *P. huashanica* has not been reported. In the present study, the wheat–*P. huashanica* derivatives H-34-8-2-6-1 and H-24-3-1-5-19-1 exhibited moderate resistance to FHB. The parent wheat 7182 was moderately susceptible whereas both H-34-8-2-6-1 and H-24-3-1-5-19-1 were moderately resistant, and thus the FHB resistance genes came from *P. huashanica*. H-34-8-2-6-1 was identified as a wheat–*P. huashanica* 1NS long arm ditelosomic addition line and H-24-3-1-5-19-1 as a wheat–*P. huashanica* 2Ns(2D) substitution line in this study.

In previous studies, the wheat–*P. huashanica* 2Ns(2D) substitution line 16-6 was highly resistant to wheat stripe rust (Du *et al.* 2015). And the wheat–*P. huashanica* 2Ns(2D) substitution line H139 enhanced wheat take-all disease resistance (Bai *et al.* 2020). In the present study, H-24-3-1-5-19-1 was also a wheat–*P. huashanica* 2Ns(2D) substitution line but it provided resistance to FHB. Thus, wheat–*P. huashanica* 2Ns(2D) substitution lines contain many disease resistance genes which need to further studied. The wheat–*P. huashanica* 1Ns addition lines 12-3 was highly resistant to brown leaf rust and the wheat–*P. huashanica* 1Ns disomic addition line H5-5-4-2 had immunity to powdery mildew at the seedling and adult stages (Du *et al.* 2014a, Han *et al.* 2020). However, wheat–*P. huashanica* 1Ns long arm ditelosomic addition line has not been identified previously. Thus, H-34-8-2-6-1 provides a new derivative material and a new germplasm resource between wheat and *P. huashanica*. Due to introduction of the whole exogenous chromosome in addition line and substitution line, the beneficial genes are often introduce along with the undesirable genes carried on the chromosome. The translocation lines carrying the target character, especially the small fragment translocation line, can be created by the EMS mutagenesis and and ^60^Co radiation of H-24-3-1-5-19-1 and H-34-8-2-6-1, which will greatly reduce the effects of the undesirable genes and increase the stability of exogenous genes in wheat.

The yield of wheat varieties bred by inter-variety hybridization decreased, and the disease resistance decreased or even disappeared (Fischer and Edmeades 2010, Li *et al.* 2008). While wild relatives of wheat carry many beneficial genes (Tester and Langridge 2010). In order to find new strategies for high yield and disease resistance, many researchers have tried to integrate disease resistance genes from wild related species into cereal cultivation background to improve wheat yield and resistance. Chromosomes or chromosome fragments can be infiltrated from one species to another by distant hybridization (Anderson 1953, Mallet 2007). Interspecific hybridization is the first step to introduce foreign variation and transfer ideal traits from wild species to cultivated species (Sharma and Gill 1983). In the early stage of the laboratory, the common wheat 7182 was used as the female parent and the *psathyrostachys huashanica* was used as the male parent for hybridization, and a heptaploid hybrid ‘H8911’ (2n = 49) was created (Chen *et al.* 1991). By backcrossing 7182 with ‘H8911’ and selfing for nine generation, 45 interspecific derivatives between wheat and *P. huashanica* were obtained in this study. In addition, the chromosomes of the hybrid F_1_ can be doubled by chromosome doubling to produce an amphidiploid. The man-made crop Triticale (*X Triticosecale* Wittmack) is an amphidiploid between wheat and rye (Gupta and Priyadarshan 1982). The amphidiploid can be further used as a bridge for the introgression of aline genes or the development of aline chromosome addition, substitution, translocation lines (Jiang *et al.* 1993).

In this study, assessed the resistance to FHB in 45 interspecific derivatives between wheat and *P. huashanica* and identified two resistant lines comprising H-34-8-2-6-1 and H-24-3-1-5-19-1. Analyses based on GISH, FISH, EST-STS, SSR, and 660K genotyping arrays confirmed H-34-8-2-6-1 as a wheat–*P. huashanica* 1Ns long arm ditelosomic addition line and H-24-3-1-5-19-1 as a 2Ns(2D) substitution line. These new FHB-resistant materials can be applied to creat small alien chromatin introgressed line in wheat breeding.

## Author Contribution Statement

JW conceived and designed the experiments. CCH, JH, LZ performed the study. LLZ and QG analyzed the date. SHL, QHY and XHC contributed new methods or models. CCH wrote the paper. All authors have reviewed drafts of the paper and approved the final draft.

## Supplementary Material

Supplemental Tables

## Figures and Tables

**Table 1.** Disease indexes and assessments of resistance and susceptibility for the 45 interspecific derivatives between wheat and *P. huashanica* and their parent common wheat 7182

Materials	2017		2018		Materials	2017		2018	
	Disease index	Judged resistances or susceptibilities evaluation	Disease index	Judged resistances or susceptibilities		Disease index	Judged resistances and susceptibilities	Disease index	Judged resistances or susceptibilities
H-34-8-2-6-1	32	MR	36	MR	H-3-5-9-3-1	78	MS	76	MS
H-210-1-1	68	MS	76	MS	H-3-7-4-2-1	58	MS	52	MS
H-1-8-1-1-2	84	HS	76	MS	H-17-7-1-1-1	56	MS	64	MS
H-3-1-1-1	46	MS	56	MS	H-30-2-3-1-1	64	MS	68	MS
H-5-9-1	52	MS	78	MS	H-2-7-8-7-7-2	72	MS	78	MS
H-8-12-2	54	MS	64	MS	H-3-3-6-3-7	46	MS	52	MS
H-9-46-1	86	HS	84	HS	H-3-5-6-3-1-9	52	MS	72	MS
H-13-4-1	96	HS	86	HS	H-17-7-1-1-8-2	62	MS	68	MS
H-19-1-1	82	HS	88	HS	H-18-1-3-1-6-4	68	MS	74	MS
H-42-3-1	48	MS	56	MS	H-20-5-1-1-3-2	74	MS	72	MS
H-20-1-1	54	MS	66	MS	H-24-4-4-1-1-3	86	HS	84	HS
H-58-1-2-1	58	MS	72	MS	H-30-4-4-1-6-4	58	MS	48	MS
H-21-10-2-2	66	MS	66	MS	H-99-1-1-2-1	72	MS	88	HS
H-45-14-1-1	64	MS	68	MS	H-48-3-2-1-7-2	64	MS	68	MS
H-37-2-1	68	MS	72	MS	H-24-3-1-6-14-3	88	HS	86	HS
H-49-4-1	86	MS	94	MS	H-48-8-2-1-1	56	MS	72	MS
H-62-1-1-1	62	MS	56	MS	H-48-8-2-1-9	68	MS	66	MS
H-19-1-1-1	78	MS	84	HS	H-3-2-1-3-5	66	MS	62	MS
H-26-1-1-1	82	HS	86	HS	H-3-2-1-3-12	92	HS	84	HS
H-24-3-1-5-19-1	38	MR	36	MR	H-3-2-2-1-1	62	MS	68	MS
H-1-11-5-1-1	82	HS	88	HS	H-3-2-3-5-1	58	MS	70	MS
H-2-4-18-7-1	48	MS	50	MS	H-3-7-4-2-2	52	MS	64	MS
H-2-4-18-7-10	56	MS	60	MS	7182	68	MS	76	MS

**Table 2.** Agronomic traits of H-34-8-2-6-1, H-24-3-1-5-19-1, and wheat parent 7182

Materials	Plant height/cm	Tiller number	Spikelength/cm	Spikelet number per spike	Kernel number per spike	Self-fertility rate/%
7182	78.56 ± 1.43 Aa	7.00 ± 1.49 Aa	8.76 ± 0.37 Aa	18.80 ± 1.23 Aab	56.50 ± 2.88 Bb	75.04 ± 1.76 Bc
H-34-8-2-6-1	76.81 ± 2.04 Aa	7.70 ± 0.95 Aa	8.90 ± 0.68 Aa	19.70 ± 1.34 Aa	66.00 ± 2.26 Aa	84.31 ± 2.36 Aa
H-24-3-1-5-19-1	67.37 ± 4.16 Bb	7.20 ± 0.92 Aa	8.60 ± 0.31 Aa	18.20 ± 1.14 Ab	56.60 ± 3.87 Bb	77.57 ± 2.59 Bb

Different capital and lower case letters indicate significant differences at p = 0.01 and 0.05.
