# Supplementary material for: Identification of resistance to Fusarium head blight and molecular cytogenetics of interspecific derivatives between wheat and Psathyrostachys huashanica
Source: Breed Sci. 2022 Jun 11;72(3):213–21. doi: 10.1270/jsbbs.21089 (PMC9653196; doi:10.1270/jsbbs.21089)
Supplement: Supplementary file 1 — Supplemental Tables [file 72_213_s1.pdf]

doi: 10.1270/jsbbs.21089

Supplemental Table 1 Classification of wheat *Fusarium* head blight symptoms.

Grade	Grading standards
0	Inoculated spikelets are not infected
1	Only inoculated spikelets are infected and no other related spikelets are infected.
2	Inoculated spikelets are infected and that their spike stalks become infected.
3	Inoculated spikelet accounts for less than 1/4 of whole spike.
4	Inoculated spikelet for more than 1/4 and less than 1/2 of whole spike.
5	Inoculated spikelet accounts for more than 1/2 of whole spike.

Supplemental Table 2 EST-STS markers used to analyze the chromosomal compositions of H-34-8-2-6-1 and H-24-3-1-5-19-1

Marker	Type	Primer (5′-3′)	Tm (°C)	Location
BE443796	STS	F:AGGCCACTCCTAAGGCAAAT<br>R:CCAGAACAGTTATGCGGGAT	60	1AL 1BL 1DL
BE497584	STS	F:CTGTTGCCAAGAGCATTGAA<br>R:GTCACAACATCATCAACCGC	60	1AL 1BL 1DL
BE446010	STS	F: GCATTTTGGAGAGAGCATCA<br>R: ATCTTTTCCATCAGCCCCTT	60	1AL 1BL 1DL
BE404332	STS	F:CATGCTGCTCAGCTTTGTGT<br>R:GCCCTCAGAGCTAATCTTGC	60	2AS 2BS 2DS
BE444851	STS	F:GGAAGATGGCCTTGTAGCTG<br>R:TCCGCACTAATGCTTTCCTCT	60	2AL 2BL 2DL
BF146221	STS	F:CTTGGAGGTGTCGTCCTTGT<br>R:CGAGTTCCAGATGCAGTACG	60	2AS 2BS 2DS
BG607805	STS	F: ACCCCTTAGAGACGCTCCAT<br>R: TAGCTCTTTGCTTGCCCAAT	60	2AL 2AS 2BS
CD452803	STS	F:TCCGCACTAATGCTTCCTCT<br>R:GCACAGGATATTAGCCAAGCA	60	2AL 2BL 2DL

Supplemental Table 3 SSR primers used to identify the chromosome substituted in H-24-3-1-5-19-1.

Marker	Type	Primer (5′-3′)	Tm (°C)	Location
Xcfd51	SSR	F:GGAGGCTTCTCTATGGGAGG<br>R:TGCATCTTATCCTGTGCAGC	60	2DS
Xwmc111	SSR	F:ATTGATGTGTACGATGTGCCTG<br>R:CATGTCAATGTCATGATGAAGC	60	2DS
Xwmc503	SSR	F:GCAATAGTTCCCGCAAGAAAAG<br>R:ATCAACTACCTCCAGATCCCGT	65	2DS
Xwmc112	SSR	F:TGAGTTGTGGGGTCTTGTTTGG<br>R:TGAAGGAGGGCACATATCGTTG	65	2DS 3AS
Xcfd160	SSR	F:CCACTACTGCGGCTAGGTCT<br>R:CTTTTCCGTGTCTCCCTAGC	60	2DL
Xgwm455	SSR	F:ATTCGGTTCGCTAGCTACCA<br>R:ACGGAGAGCAACCTCCC	55	2DS 6BL
wmc41	SSR	F:TCCCTCTTCCAAGCGCGGATAG<br>R:GGAGGAAGATCTCCCGGAGCAG	60	2DL
wmc167	SSR	F:AGTGGTAATGAGGTGAAAGAAG<br>R:TCGGTCGTATATGCATGTAAAG	50	2DL 2BL
barc168	SSR	F:GCGATGCATATGAGATAAGGAACAAATG<br>R:GCGGCTCTAAGGCGGTTTCAAAT	50	2DS
Xgwm210	SSR	F:TGCATCAAGAATAGTGTGGAAG<br>R:TGAGAGGAAGGCTCACACCT	60	2AS 2BS 2DS
Xgwm261	SSR	F:CTCCCTGTACGCCTAAGGC<br>R:CTCGCGCTACTAGCCATTG	55	2DS
Xgwm484	SSR	F:ACATCGCTCTTCACAAACCC<br>R:AGTTCCGGTCATGGCTAGG	55	2DS
